# What shapes ground beetle assemblages in a tree species-rich subtropical forest?

**DOI:** 10.3897/zookeys.1044.63803

**Published:** 2021-06-16

**Authors:** Pascale Zumstein, Helge Bruelheide, Andreas Fichtner, Andreas Schuldt, Michael Staab, Werner Härdtle, Hongzhang Zhou, Thorsten Assmann

**Affiliations:** 1 Institute of Ecology, Leuphana University Lüneburg, Universitätsallee 1, 21335 Lüneburg, Germany Leuphana University L&uuml;neburg Germany; 2 Institute of Biology/Geobotany, Martin Luther University Halle-Wittenberg, 06108 Halle (Saale), Germany Martin Luther University Halle Germany; 3 German Centre for Integrative Biodiversity Research (iDiv) Halle-Jena-Leipzig, 04103 Leipzig, Germany German Centre for Integrative Biodiversity Research Leipzig Germany; 4 Forest Nature Conservation, Georg-August-University Göttingen, Büsgenweg 3, 37077 Göttingen, Germany Georg-August-University G&ouml;ttingen Germany; 5 Ecological Networks, Technical University Darmstadt, Schnittspahnstraße 3, 64287 Darmstadt, Germany Technical University Darmstadt Darmstadt Germany; 6 Key Laboratory of Zoological Systematics and Evolution, Chinese Academy of Sciences, 1 Beichen West Road, Chaoyang District, Beijing 100101, China Key Laboratory of Zoological Systematics and Evolution, Chinese Academy of Sciences Beijing China

**Keywords:** Abundance, BEF-China, biomass, canopy cover, Carabidae, elevational gradient, herb cover, pH-value, species richness

## Abstract

As woody plants provide much of the trophic basis for food webs in forests their species richness, but also stand age and numerous further variables such as vegetation structure, soil properties and elevation can shape assemblages of ground beetles (Coleoptera: Carabidae). However, the combined impact of these numerous variables on ground beetle diversity and community structure has rarely been studied simultaneously. Therefore, ground beetles were studied in 27 plots in a highly diverse and structurally heterogeneous subtropical forest ecosystem, the Gutianshan National Park (southeast China) using pitfall traps and flight interception traps. Both trapping methods collected partly overlapping species spectra. The arboreal fauna was dominated by lebiines and to a smaller extent by tiger beetles and platynines; the epigeic fauna comprised mostly representatives of the genus *Carabus* and numerous tribes, especially anisodactylines, pterostichines, and sphodrines. Ground beetle species richness, abundance, and biomass of the pitfall trap catches were analyzed with generalized linear mixed models (GLMMs), fitted with seven environmental variables. Four of these variables influenced the ground beetle assemblages: Canopy cover, herb cover, pH-value of the topsoil and elevation. Contrary to our expectations, woody plant species richness and stand age did not significantly affect ground beetle assemblages. Thus, ground beetles seem to respond differently to environmental variables than ants and spiders, two other predominantly predatory arthropod groups that were studied on the same plots in our study area and which showed distinct relationships with woody plant richness. Our results highlight the need to study a wider range of taxa to achieve a better understanding of how environmental changes affect species assemblages and their functioning in forest ecosystems.

## Introduction

Tropical and subtropical forests are among the world´s ecosystems with the highest biodiversity. A substantial part of this biodiversity consists of arthropods, which are involved in many ecosystem processes (Basset et al. 2012; Schuldt et al. 2015b; Stork 2018). Numerous biotic and abiotic variables can influence their diversity, but also the abundance and biomass of these arthropods (Rainio and Niemela 2003; Mottl et al. 2020; Marrec et al. 2021). Trees structure forest ecosystems and, as they are the most important primary producers of biomass, they provide the basis for much of the food web consisting of decomposers, herbivores, and predators. As many biodiversity patterns in forest ecosystems seem to be influenced by bottom-up processes (Gruner 2004; Wu et al. 2013; Korboulewsky et al. 2016), woody plant species richness can have a strong impact on higher trophic levels (Schuldt et al. 2017). As a consequence, the diversity of herbivores may be strongly determined by the diversity of trees (Riihimaki et al. 2005; Wang et al. 2019). Also at higher trophic levels (predators, parasitoids), there seem to be bottom-up effects from woody plant diversity (Strong et al. 1984; Lefcheck et al. 2015; Schuldt et al. 2015a). However, such relationships between plant diversity and arthropods are not always present or apparent (Vehvilainen et al. 2008).

To analyze the effects of woody plant species richness on arthropod diversity in a real-world forest ecosystem, it is important to consider that environmental variables can also influence communities or assemblages of arthropods (Thiele 1977; Bonn and Schröder 2001). In experiments with manipulated woody plant species richness, possible biodiversity effects can be better determined (Loreau et al. 2001; Duffy 2009); however, conclusions from such manipulative experimental approaches cannot simply be transferred to forests with long-lasting maturation processes, in particular because experiments are usually not much older than a few decades. Therefore, real-world ecosystems are of particular importance for the study of arthropod communities (or assemblages) in forests of different stand ages.

Ground beetles (Coleoptera: Carabidae) represent a highly diverse predatory taxon and are typical of forest ecosystems across latitudinal gradients (Zou et al. 2013; Nolte et al. 2017; Garcia-Tejero et al. 2018). Moreover, ground beetles in temperate and boreal regions are known to be sensitive to numerous edaphic variables (e.g., pH-value: Paje and Mossakowski 1984; moisture: Antvogel and Bonn 2001). Even effects of land-use legacy, tree age, vegetation structure and succession are known (Terrell-Nield 1990; Assmann 1999; Jelaska et al. 2011; Marrec et al. 2021). In general, ground beetles are considered as a well-known taxon for bio-indication since numerous species react sensitively to small changes in environmental variables (Koivula 2011).

The shaping variables of ground beetle assemblages in global biodiversity forest hotspots are poorly understood. In particular, the ground beetles of the southeast Chinese subtropics with summer rainfalls, which host particularly species-rich forests (Liu et al. 2003; Tang 2006), provide an interesting opportunity for study. We worked in Gutianshan National Park (Zhejiang, southeast China) that covers an area with remarkable subtropical tree and shrub species diversity. Relationships between biodiversity and ecosystem functions and the effects of environmental variables have been studied extensively in this area (e.g., Schuldt et al. 2010; Lang et al. 2012; Staab et al. 2014; Eichenberg et al. 2015; Zou et al. 2015; O’Brien et al. 2016; Brezzi et al. 2017). However, while the main abiotic and biotic drivers potentially shaping herbivore, decomposer and predator diversities have been studied (e.g., Schuldt et al. 2011; Staab et al. 2014; Schuldt et al. 2015a; Binkenstein et al. 2018; Staab et al. 2021) studies on ground beetles are still lacking for this National Park.

A study of ground beetles would reveal not only how this arthropod taxon responds to environmental variables, but the comparison with other important taxa (esp. spiders and ants) could provide a better understanding of how environmental variables affect the abundance and species diversity of predatory arthropods. Thus, since environmental variables can potentially influence ground beetle assemblages, we hypothesize that abundance, richness, and biomass of ground beetles increases with (H1) woody plant species richness and (H2) stand age in a subtropical forest in China. Moreover, we expect that (H3) the structural richness of vegetation and abiotic variables affect ground beetle abundance, richness, and biomass. Specifically, we investigate whether woody plant species richness, stand age of forests plots, structural richness of vegetation (cover of canopy and herb layer), and abiotic variables (elevation and soil pH) influence abundance, species richness, and biomass of ground beetles.

## Material and methods

### Study area and plots

Gutianshan National Park (Gutianshan NP), formerly Gutianshan National Nature Reserve, is located in the western part of Zhejiang Province in southeast China (29°14'N, 118°07'E). The park is approximately 81 square kilometers in size and was established in 1975, first as a National Forest Reserve, to preserve parts of the old‐growth evergreen broad‐leaved forest in the region. The climate is typical for subtropical areas with an annual mean temperature of 15.3 °C and ~2000 mm mean precipitation per year, occurring mostly between March and September (Yu et al. 2001). Further meteorological information is provided by Bruelheide et al. (2011) or cited therein. The park is characterized by a mixed broad-leaved forest composed of deciduous and evergreen tree species, and in which *Castanopsis eyrei* (Champion & Bentham, 1845) and *Schima superba* (Gardner & Champion, 1849) are the prevailing tree species (Hu and Yu 2008; Legendre et al. 2009). Most stands consist of secondary forest, with maximum ages of trees ≤ 180 years. The park is located in a mountain range with elevations varying between 300 and 1260 m above sea level. Our study focused on stands ranging from 251 to 903 m a.s.l. The local rock is granitic and thus the topsoil pH-values ranges from 5.5 to 6.5 (Bruelheide et al. 2011).

In 2008, 27 study plots, each measuring 30 m × 30 m, were established by a research consortium of Chinese and European scientists (Bruelheide et al. 2011). The plots were randomly distributed throughout the park, excluding sites with slopes > 55°. The plots included gradients of woody plant species richness (25–69 trees and shrub species per plot) and stand age (21–115 years). Further information on plot characteristics and study design is provided by Bruelheide et al. (2011).

### Pitfall trapping

We sampled ground beetles using four pitfall traps in each plot, installed in the corners of the central 10 m × 10 m square (resulting in 108 traps). Traps were plastic cups with a diameter of 8.5 cm and a capacity of ~550 ml filled with 150 ml of preserving solution (40% ethanol, 20% glycerol, 10% acetic acid, 30% water). They were open continuously during the main vegetation period in 2009 (end of March to the beginning of September). Traps were emptied fortnightly (in total ten collections per trap) and catches were preserved in 70% ethanol until identification (Schuldt et al. 2011). Flight interception traps were installed close to the pitfall traps in 2010 (Schuldt et al. 2015a; Staab et al. 2021). Ground beetles were sorted and body length was measured for biomass calculation (see section *Statistical analysis*). Specimens were identified to species level or classified to morphospecies by carabid taxonomists (David W. Wrase, Thierry Deuve, and Thorsten Assmann). Results from such a “morphospecies approach” show high correlation to true species diversity (Oliver and Beattie 1996). Hereafter, we use the term species for both diagnosed species and morphospecies to characterize our catches.

### Environmental data

During plot establishment, a comprehensive set of environmental variables of biotic and abiotic habitat characteristics was collected (Bruelheide et al. 2011). We asked if seven variables, known to influence ground beetles assemblages (e.g., Assmann 1999; Antvogel and Bonn 2001; Marrec et al. 2021) could predict the abundance, species richness and biomass of ground beetles. The age of the forest included in each plot was estimated using stem core measurements of the tree with the fifth largest diameter at breast height within each plot. Woody plant species richness was determined as the number of all tree and shrub species represented by individuals larger than 1 m in the plot (Bruelheide et al. 2011). Species richness and abundance of the herbaceous layer were estimated for all herb species smaller than 1 m height in the inner 10 m × 10 m subplots (Both et al. 2011). Canopy cover (%), herb cover (%), elevation (meters a.s.l.), and pH-value of the topsoil (0–5 cm; taken from nine dried and sieved soil samples per plot, pooled and measured potentiometrically) were additionally assessed as plot characteristics (see Bruelheide et al. (2011) for details).

### Statistical analysis

All statistical analyses were conducted using the packages glmmTMB (Brooks et al. 2017) and DHARMa (Hartig 2020) in R 3.6.3 (http://www.R-project.org). We applied generalized linear mixed models (GLMMs) to assess the effects of biotic (woody species richness, stand age, canopy and herb cover, herb species richness) and abiotic (topsoil pH-value, elevation) stand conditions (i.e., plot characteristics) on the abundance, species richness and biomass of ground beetles. Plot identity was used as a random factor to account for the nested data structure (traps nested in study plots). Abundance and species richness data were modelled with a Poisson distribution and log-link function. There was no indication of overdispersion in the data for either abundance (p = 0.25) or species richness (p = 0.20). For the biomass data, we applied a Gamma distribution with a log-link and added a value of 0.01 to the observed biomass prior to model fitting to ensure the convergence of the algorithm. Ground beetle biomass was calculated according to the following formula:

ln *y* = -8.92804283 + 2.5554921 ln *x*,

where: *x* is the measured body length of the specimen and *y* is the estimated body weight of the individual (Schwerk and Szyszko 2007). The calculated biomass estimates were then summed over all individuals per plot.

Model selection was based on likelihood-ratio tests starting with a fully saturated model that included all predictors and the interaction between woody species richness and stand age to test, if possible, species richness effects depended on the age of the forest plots. We sequentially removed non-significant (p > 0.05) terms and tested for assumptions of the best-fitting model following (Zuur et al. 2009). Prior to analysis, all predictor variables were standardized (mean = 0, SD = 1) and tested for critical correlations (all variance inflation factors were < 1.75).

## Results

### Number of species, abundance, and biomass

In total, we caught 258 ground beetle specimens in the pitfall traps, belonging to 22 species (Table 1). Catches ranged from two to 27 specimens per plot (mean 9.6 ± 1.19 SE), based on a relatively low number of individuals (2.4 ± 0.22) per trap. As expected, species richness was strongly correlated with abundance (Pearson correlation coefficient r = 0.85, p ≤ 0.001). Biomass of captured ground beetles ranged from 0.15 to 10.63 g per plot. The average biomass per plot was 3.11 g (± 0.54 SE).

**Table 1.** Collected ground beetles from pitfall traps of 27 plots in Gutianshan NP. For classification, we followed the systematics of the Palearctic catalogue (Löbl and Löbl 2017). Abundance (No.) and body size (mean body size if more than one individual caught) is given for each (morpho-) species. The elevation data refer to the highest and lowest plots where the ground beetles were caught.

Tribe	(Morpho-) Species	No.	Size (mm)	Elevation (m a.s.l.)
Anisodactylini	undet. spec. 1	60	8.9	348–903
	undet. spec. 2	3	8	639
Carabini	Carabus (Apotomopterus) davidis Deyrolle, 1878	5	35	566–679
	Carabus (Isiocarabus) kiukiangensis Bates, 1888	20	30	348–903
	Carabus (Damaster) lafossei Feisthamel, 1845	5	42	566–679
Brachinini	Pheropsophus (Stenaptinus) beckeri Jedlicka, 1930	1	14	647
Harpalini	*Amara* spec. 1	1	8	542
	*Harpalus* spec. 1	1	12	617
Lebiini	Calleida (Callidiula) spec. 1	1	12	617
	*Lachnoderma asperum* Bates, 1883	1	8	880
	Pericalina, undet. 1	1	5	617
Pentagonicini	*Pentagonica* spec. 1	10	4.5	251–679
	*Pentagonica* spec. 2	1	5	542
Perigonini	*Perigona* spec. 1	4	3	542–720
Pterostichini	*Lesticus* spec. 1	7	25.3	590–903
	*Lesticus* spec. 2	43	28.1	251–903
	*Pterostichus* spec. 1	47	24.8	251–903
	*Pterostichus* spec. 2	3	11	419–670
Sphodrini	*Synuchus* spec. 1	32	13.7	251–679
	*Synuchus* spec. 2	10	10.9	251–903
Cicindelini	Cylindera (Ifasina) kaleea Bates, 1863	1	9	880
Collyridini	*Tricondyla macrodera* Chaudoir, 1861	1	19	566

The number of ground beetle specimens in flight interception traps was lower than in pitfall traps. In total, we caught 49 individuals of six species (Table 2), three of which were recorded also by the pitfall traps on the forest floor. As we caught few ground beetles in the flight interception traps, we performed further analyses only with the catches of the pitfall traps. Although there was overlap in species spectra, the catches of the two trapping methods differed strongly. Species of the genus *Carabus* and the tribes Anisodactylini and Pterostichini were caught exclusively in the pitfall traps; and lebiines were more abundant in the flight interception traps (Tables 1, 2; Fig. 4).

**Table 2.** Collected ground beetles from flight interception traps of 27 plots in Gutianshan NP. For classification, we followed Löbl and Löbl (2017). Abundance (No.) and body size (mean body size if more than one individual caught) given for each (morpho-) species.

Tribe	(Morpho-) Species	No.	Size (mm)
Lebiini	*Lachnoderma asperum* Bates, 1883	1	8
	*Lioptera erotyloides* Bates, 1883	1	13
	Coptodera (Coptoderina) spec. 1	42	7.5
Pentagonicini	*Pentagonica* spec. 1	2	4.5
Platynini	undet. spec. 1	1	8
Collyridini	*Tricondyla macrodera* Chaudoir, 1861	2	19

### Ground beetle assemblages and environmental variables

**Figure 1.**
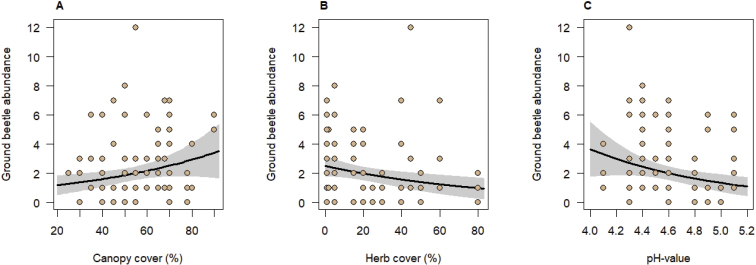
Relationships between ground beetle abundance and canopy cover (**A**), herb cover (**B**) and pH-value of the soil (**C**). Black lines indicate significant relationships at p < 0.05 obtained from mixed-effects models (keeping other significant predictors fixed at their means) with grey areas indicating the 95% confidence intervals. Points represent observed values per trap. Note that some traps had similar abundance and predictor values. The fixed-effects explained 22% of the variation in ground beetle abundance.

**Figure 2.**
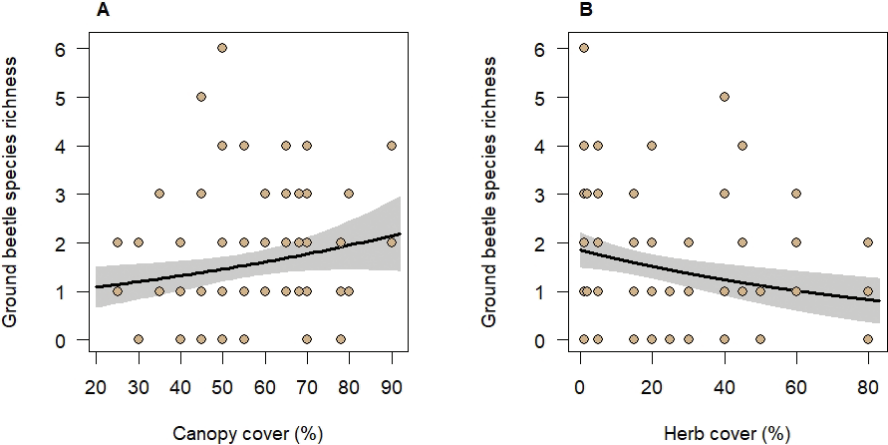
Relationships between ground beetle species richness and canopy cover (**A**) and herb cover (**B**). Black lines indicate significant relationships at p < 0.05 obtained from mixed-effects models (keeping other significant predictors fixed at their means) with grey areas indicating the 95% confidence intervals. Points represent observed values per trap. Note that some traps had similar richness and predictor values. The fixed-effects explained 12% of the variation in ground beetle species richness.

**Figure 3.**
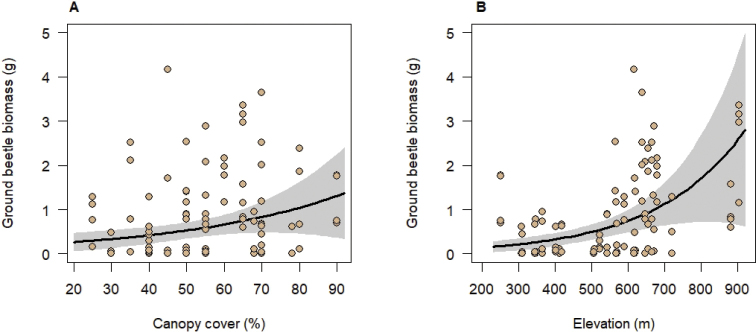
Relationships between ground beetle biomass and canopy cover (**A**) and herb cover (**B**). Black lines indicate significant relationships at p < 0.05 obtained from mixed-effects models (keeping other significant predictors fixed at their means) with grey areas indicating the 95% confidence intervals. Points (slightly jittered to improve visibility) represent observed values per trap. The fixed-effects explained 30% of the variation in ground beetle biomass.

Four of the seven environmental variables tested for effects on ground beetles in generalized linear mixed models were significantly related to the ground beetle assemblages. Canopy cover had a positive influence on species richness, abundance, and biomass of ground beetles (Table 3, Figs 1A, 2A, 3A); however, herb cover influenced ground beetle species richness and abundance negatively (Table 3, Figs 1B, 2B). In addition, ground beetle abundance decreased significantly with increasing soil pH (Table 3, Fig. 1C). Finally, ground beetle biomass significantly increased with elevation (Table 3, Fig. 3B). This is related to the presence of *Carabus* species, the ground beetles with the largest body lengths in our study, especially at higher elevations. The biomass of representatives of the genus *Carabus* was strongly correlated with elevation (Pearson correlation coefficient r = 0.65, p ≤ 0.001). The biomass of the representatives of the tribes Pterostichini, which included species with the second largest individuals, however, was not significantly correlated with elevation (r = 0.26, p > 0.05). The same was true for the Anisodactylini (r = 0.005, p > 0.05) which were numerous in terms of individuals (n = 63). Neither species richness of woody plants and of herbs nor stand age significantly influenced any characteristic of the ground beetle assemblages (Table 3).

**Figure 4.**
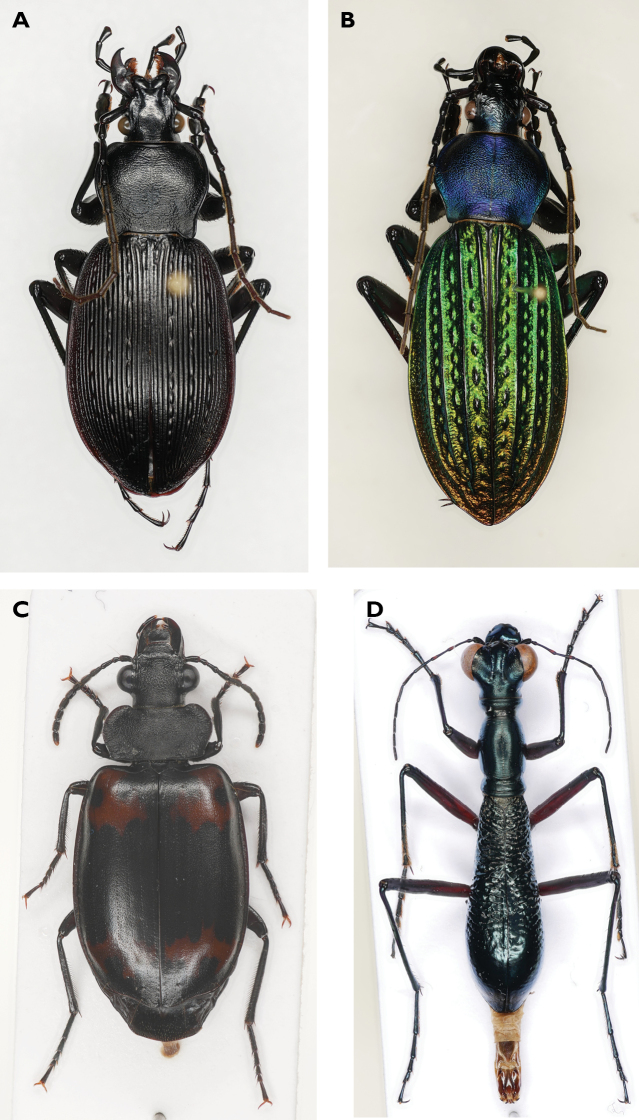
Representatives of ground beetles from pitfall traps and flight interception traps in Gutianshan NP **A***Carabus kiukiangensis***B***Carabus davidis***C***Lioptera erotyloides***D***Tricondyla macrodera*.

**Table 3.** Results from mixed-effects models for ground beetle abundance, species richness, and biomass. P-values were obtained from likelihood-ratio tests starting with a full-saturated model and removing non-significant (p > 0.05) terms sequentially. Significant predictors (p < 0.05) are indicated in bold.

	Abundance		Species richness		Biomass	
	χ²	p-value	χ²	p-value	χ²	p-value
Woody species richness (WSR)	0.07	0.787	0.04	0.849	1.44	0.230
Stand age	0.33	0.565	0.00	0.961	1.17	0.280
Canopy cover	4.28	**0.039**	3.90	**0.048**	4.98	**0.026**
Herb cover	4.67	**0.031**	5.98	**0.014**	0.60	0.438
Herb species richness	1.60	0.206	0.89	0.345	0.18	0.673
pH-value (topsoil)	5.30	**0.021**	3.64	0.056	0.97	0.324
Elevation	0.39	0.531	0.18	0.668	14.71	**<0.001**
WSR * stand age	0.01	0.941	0.19	0.664	2.41	0.120

## Discussion

Our study revealed that four environmental variables impacted on ground beetle assemblages at our sample sites. Contrary to our expectations, woody plant species richness (H1) and stand age (H2) did not influence ground beetle assemblages in this study. We did corroborate H3, as higher canopy cover led to increased species richness, abundance, and biomass of ground beetles, and because ground beetle abundance and species richness decreased with higher herb cover. Moreover, soil pH negatively influenced ground beetle abundance, and greater biomass of beetles was found at higher elevations.

### Vegetation effects and ground beetle assemblages

Classical ecological theory such as the “enemies” hypothesis (Root 1973; Staab and Schuldt 2020), predict higher predator abundance and diversity with increasing plant diversity caused by different mechanisms at herbivore and predator trophic levels. This theory has been corroborated in other studies, especially in non-forest ecosystems (Andow 1991; Haddad et al. 2009). Furthermore, Jouveau et al. (2020) demonstrated positive and additive effects of vegetation diversity (understory, canopy and surrounding scales) on the density of ground beetles in a tree diversity experiment with planted tree individuals. Nonetheless, with increasing diversity of an ecosystem, mixed results are often obtained. For example, some studies postulate positive relationships between plant diversity and ground beetles, while other studies show no or negative relationships (Vehvilainen et al. 2008; Worthen and Merriman 2013; Yeeles et al. 2017; Zou et al. 2019).

We found no relationship between woody plant species richness and species numbers, abundance, or biomass of ground beetles in Gutianshan NP. This result is consistent with those from numerous other studies on plants and arthropods, especially ground beetles (Wolters et al. 2006; Harry et al. 2019; Corcos et al. 2021). Ground-dwelling predators do not directly depend on vegetation, while most herbivores depend directly on host plant selection. This difference alone may result in different patterns for the two trophic groups. It is primarily the structural features of vegetation that increase with increasing woody plant species diversity (Bruelheide et al. 2011; Schuldt et al. 2019) that would be expected to affect predator assemblages. Such structural patterns, in turn, influence microclimatic factors such as temperature, humidity, and light availability and thus the activity and distribution of species such as carabid beetles (Work et al. 2011). Nevertheless, the non-significant results of the mixed-effects model provide no support for relationships between woody plant species richness and ground beetle assemblages in Gutianshan NP. However, previous studies of epigeic arthropods in our study plots found mixed evidence of relationships with woody plant species richness. The relationship for spiders was negative but positive for predatory ants, with no relationship for omnivorous ants (Schuldt et al. 2011; Staab et al. 2014).

The positive relationship between woody plant and ground beetle diversity found by Zou et al. (2019) is limited to mature forests in a study area in temperate China. For secondary forests, no correlation was found, which the authors attributed to the lower forest age. The forest in Gutianshan NP is classified as secondary forest, because it was previously used agriculturally (Bruelheide et al. 2011). Compared to the forests studied by Zou et al. (2019), the older plots in Gutianshan NP closely resemble mature stands. However, we found no relationship between ground beetle richness and woody plant species diversity in older stands in Gutianshan NP. Possibly, these forest plots are not old enough to have fostered a reasonably distinct natural ground beetle community.

Greater closure of the canopy layer was associated with more beetle species and specimens. However, our results contrast findings from numerous studies in forests of boreal, temperate and Mediterranean climate zones, according to which the number of ground beetle species increases with decreasing canopy cover (Koivula et al. 2002; Taboada et al. 2008; Thorn et al. 2016). These findings can be explained (at least partly) by the presence of more open habitat species of ground beetles in addition to the forest species in open or structural-rich forest sites (Heliölä et al. 2001; Magura et al. 2001). Nonetheless, if open habitat specialists are rare in the study region and simultaneously forest specialists avoid more open patches in Gutianshan NP, beetle catches would be lower in plots with less canopy cover. Although the hypothesis is tempting, habitat preferences of ground beetles in Chinese forests and other habitats are not as well studied as in the western Palearctic (Yu et al. 2009; Zou et al. 2015) and no data are available to test this idea.

In contrast, herb cover had a negative influence on ground beetle abundance and species richness. Studies from forests in both temperate and Mediterranean climate regions have documented both negative and positive influences of the forest herb layer on the species richness of ground beetles (Antvogel and Bonn 2001; Taboada et al. 2010; Liu et al. 2016). It is known that the cover of the herb layer can impede the movements of ground beetles on the forest floor (Taboada et al. 2010). Thus, a denser understory could hinder ground beetle activity within these stands. This, in turn, could negatively affect hunting processes in such stands. It might also reduce estimates of abundance and diversity that depend on activity-based trapping as in our study which uses pitfall traps. Contrastingly, a greater herb cover provides protection from potential predators. Additionally, the forest understory can modify the microclimate (temperature, humidity, sun-exposition), which is known to shape the ecological niches of many ground beetles (Thiele 1977; Bonn and Schröder 2001). Nonetheless, the influence of understory vegetation could be more complex and therefore requires further research with regard to ground beetles.

### Abiotic effects and ground beetle assemblages

The guild of predatory arthropods (Araneae, Chilopoda, Formicidae, cavity-nesting wasps and their parasitoids) in Gutianshan NP showed a significant decrease of both abundance and species richness with increasing elevation (Binkenstein et al. 2018). In contrast, we found that the biomass of the predominately predatory ground beetles studied on the same plots increased along the same elevational gradient. Therefore, response of organisms to elevation depends not only on trophic guild (predators, herbivores, decomposers) in these forests (Binkenstein et al. 2018), but it can also vary within trophic guilds.

Our results for biomass indicate that beetle biomass within plots increases with elevation. This relationship is driven by the higher numbers of representatives of the genus *Carabus* (Fig. 4A, B), with specimens up to 42 mm in body length, the largest ground beetles in our study. The genus *Carabus* does not occur in the tropics and only a few species occur in the summer humid subtropics (such as in Gutianshan NP). The centers of species diversity are clearly in the temperate climatic zones (Breuning 1932; Meyer 2008). The general climate preference of *Carabus* species may therefore be reflected in our catches, where we found more individuals of these species at the cooler (and moister) higher elevations.

Ground beetles are able to adapt to a wide range of varying pH-values (Krogerus 1939, 1960; Paje and Mossakowski 1984); however, our data showed significantly negative relationship between ground beetle abundance and increasing topsoil pH. Evidence from field studies suggests that different ground beetle species prefer different pH-values in the soil (Mossakowski 1970; Matern et al. 2007). Explanations have been suggested only for a very limited number of species (e.g., species adapted to feed on snails, which prefer calcerous soils with higher pH-values: Assmann 2003). To our knowledge, no such studies are available for ground beetles from China. However, the pH-gradient we found in Gutianshan NP is relatively compressed, and also indirect effects, e.g., via plants, could influence ground beetle assemblages.

### Low species number, abundance, and biomass of ground beetles

The overall number of ground beetle specimens, species and biomass was relatively low in Gutianshan NP. Given the fact, that our study took place in a subtropical hotspot of vascular plant diversity, we expected higher numbers of ground beetles. For example, studies using the same sampling approach in Central European forests have reported 60 times more ground beetle specimens and 90 times more ground beetle biomass (Homburg et al. 2019; Hülsmann et al. 2019). Similarly, the number of species from temperate forests is higher (Nolte et al. 2017; Marrec et al. 2021). Further south, towards the European subtropical zone, Brandmayr et al. (1983) found similar low catches of both individuals and species, but in a less plant diverse forest (Mediterranean forest dominated by *Quercus ilex* (Linnaeus, 1753)). Comparable subtropical forest studies in China are largely lacking, but low catch rates of ground beetles have also been reported from another study conducted in Gutianshan NP (Yu et al. 2017).

Pitfall trap catches for carabids seem to be low in most tropical evergreen forests (Vennila 1999, 2000; Maveety et al. 2011; Qodri et al. 2016). In comparison to Gutianshan NP, the dry tropics of Colombia host more species and greater abundance (Ariza et al. 2021). Many of these tropical species occur mostly in the canopy. Terry Erwin was the pioneer of research on insect diversity of this forest stratum (Erwin 1982a, b, 2002), discovering many new species in the canopy of South American rainforests (Arndt et al. 2001; Erwin 2004a, b). However, we know from other studies that the ground strata can contribute to the overall biodiversity similar to that of higher strata, such as canopies (Stork and Grimbacher 2006).

### Catches from flight interception traps

Although only a few species were recorded in our flight interception traps, they represent guilds or taxonomic entities that are well known from the tropics (Erwin and Erwin 1976; Erwin 1982b, 1983; Maveety et al. 2011), illustrating that the fauna of the subtropical Gutianshan NP is composed of temperate and tropical elements. At least some of these species have morphological adaptations to an arboreal life like pectinate claws and adhesive setae on the lower side of the tarsi (Erwin 1979; Stork 1980). All species from our flight interception traps show these morphological adaptations: lebiine species with their ectoparasitic larval development (e.g., *Lioptera erotyloides* (Fig. 4C), which mimics erotylids in coloration, its possible hosts which also occur in Gutianshan NP (cf. Erwin and Erwin 1976; Hunting and Yang 2019)), the collyridine *Tricondyla macrodera* (Fig. 4D), which not only resembles ants in their habitus, but also regularly hunts them (Shook and Wu 2007; own observations), and the platynine species (cf. Whitehead and Ball 1997).

The epigeic assemblages with their few species and individuals, but also the arboreal fauna with its specific morphological adaptations in Gutianshan NP resembles the ground beetle fauna typical for tropical forests. However, further study is required to achieve a better understanding of patterns of ground beetle species richness in subtropical forests.
